# Increased Neural Habituation in the Amygdala and Orbitofrontal Cortex in Social Anxiety Disorder Revealed by fMRI

**DOI:** 10.1371/journal.pone.0050050

**Published:** 2012-11-29

**Authors:** Ronald Sladky, Anna Höflich, Jacqueline Atanelov, Christoph Kraus, Pia Baldinger, Ewald Moser, Rupert Lanzenberger, Christian Windischberger

**Affiliations:** 1 MR Centre of Excellence, Medical University of Vienna, Vienna, Austria; 2 Center for Medical Physics and Biomedical Engineering, Medical University of Vienna, Vienna, Austria; 3 Department of Psychiatry and Psychotherapy, Medical University of Vienna, Vienna, Austria; 4 Department of Psychiatry, University of Pennsylvania Medical Center, Philadelphia, Pennsylvania, United States of America; University of Massachusetts Medical School, United States of America

## Abstract

A characterizing symptom of social anxiety disorder (SAD) is increased emotional reactivity towards potential social threat in combination with impaired emotion and stress regulation. While several neuroimaging studies have linked SAD with hyperreactivity in limbic brain regions when exposed to emotional faces, little is known about habituation in both the amygdala and neocortical regulation areas. 15 untreated SAD patients and 15 age- and gender-matched healthy controls underwent functional magnetic resonance imaging during repeated blocks of facial emotion (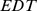) and object discrimination tasks (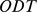). Emotion processing networks were defined by a task-related contrast (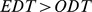). Linear regression was employed for assessing habituation effects in these regions. In both groups, the employed paradigm robustly activated the emotion processing and regulation network, including the amygdalae and orbitofrontal cortex (OFC). Statistically significant habituation effects were found in the amygdalae, OFC, and pulvinar thalamus of SAD patients. No such habituation was found in healthy controls. Concurrent habituation in the medial OFC and the amygdalae of SAD patients as shown in this study suggests intact functional integrity and successful short-term down-regulation of neural activation in brain areas responsible for emotion processing. Initial hyperactivation may be explained by an insufficient habituation to new stimuli during the first seconds of exposure. In addition, our results highlight the relevance of the orbitofrontal cortex in social anxiety disorders.

## Introduction

According to recently published epidemiological data, social anxiety disorder (SAD) has a 12-month prevalence rate of 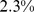 in Europe [1], 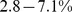 in the USA [2], and 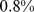 in Japan [3] (for data on other regions refer to [4]). SAD is a disabling condition impairing normal social life as patients tend to limit or remove themselves from social situations where they may be subject to evaluation by other people. This avoidance behavior is based on the fear to display anxiety symptoms (e.g., blushing) or act in a way (e.g., stuttering) that will be humiliating or embarrassing and potentially lower their social status and acceptance. If evasion is not possible, such situations are endured with intense anxiety or distress, comparable to the symptomatic of panic attacks. Although patients commonly recognize their fear as excessive or unreasonable, their behavior has devastating consequences for their social relationships, career opportunities, family life, and partner relations. SAD entails not only personal hardships for patients and their families but also, as a consequence, enormous economic and social burden. Furthermore, SAD during adolescence or young adulthood is an important predictor of subsequent alcohol and cannabis dependence [2], [5] and depressive disorders [6]. In particular, SAD might be considered a risk factor for major depression, given the commonly earlier onset of SAD in co-morbid patients [7].

SAD has been linked to negatively biased appraisals and cognitive interpretations of social interactions [8] causing the misconception of harmless situations as threat to their status within a social group. Consequently, repeated biased perception of social cues can establish a distorted belief in the patients' own social competences (e.g., being incapable of proper social interactions) and the interpretation of the behavior of others (e.g., being constantly judged and taunted by others) [9].

Besides this biased social cognition, brain networks for emotion and stress regulation [10] are assumed to be disrupted in SAD and other anxiety disorders [11]–[13]. Determining the nature and intensity of emotions critically depends on both the initial appraisal and subsequent neuronal feedback to saliently presented emotional stimuli [14]. Accordingly, emotion processing may be described as a three-step procedure: (1) identification of emotional significance, (2) change of the affective mental state in response to the stimulus and, most importantly, (3) regulation of the emotional state according to a desired behavioral state [15].

The limbic system, particularly the amygdala, receives input from multiple sensory modalities. The amygdala has been consistently identified as a core hub or gateway for emotion processing, identification, and evaluation of the affective value of a stimulus [16], [17]. The function of the amygdala is central in the recognition of threat [18] and beyond that it is a central processing hub for decision making in unpredictable and ambiguous situations [19]. In social interactions, the amygdala is required for the processing of facial display of emotions [20]–[23]. Lesions in this brain area may lead to significant changes in social behavior and defects in emotion recognition [24]. Compared to healthy individuals, anxiety disorder patients, and SAD patients in particular, have shown amygdalar hyperactivation when confronted with emotional faces of situations that might entail social threat [25]–[28]. In a quantitative meta-analysis of functional neuroimaging studies evaluating hyper- and hypoactivation in patients with anxiety disorders, hyperactivity of the amygdala was identified as a shared neurobiological feature in SAD, specific phobia and post-traumatic stress disorder (PTSD) [29].

The amygdala maintains strong anatomical and functional connections to regions within the prefrontal cortex that mediate emotion regulation in regard to contextual information, long-term consequences, and voluntary emotion regulation [30]–[33]. Social animals with prefrontal lesions are known to lose their position or membership in a group hierarchy [34]. Previous studies have highlighted activation differences in the orbitofrontal cortex (OFC) or the ventromedial PFC (vmPFC) for (social) anxiety disorder patients [25], [35]–[37] and in anxiety-prone non-clinical subjects [26], [38]. Furthermore SAD patients exhibit decreased resting-state connectivity [39]–[41] and structural connectivity [42]–[44].

While the the amygdala-prefrontal network may be well-described as a necessary neuronal correlate for emotion processing and previous studies have highlighted activation abnormalities in anxiety disorders, less is known about the exact neuronal mechanisms and their temporal dynamics. Based on the specific symptoms of SAD, it may be hypothesized that these patients fail to adequately down-regulate amygdala activation when in an anxiety-provoking situation [13], [45]. However, the question remains on whether the inability to engage this down-regulation mechanism is a persistent and obligatory feature of SAD whenever exposed to social stress or emotional (potentially intimidating and judgmental) faces.

One key regulation mechanism in the nervous system is habituation, which can be understood as a diminished response of a single nerve cell or a larger neuronal population to a repeatedly presented stimulus [46]. While the BOLD response of the amygdala towards fearful faces is assumed to be stable over multiple scan sessions [47], within-run habituation has been observed for fear-inducing faces [48]–[51] and threat cues [52] in the amygdala and the PFC [53] in healthy subjects. The particular importance of habituation in the amygdala and associated brain regions for the pathophysiology of social anxiety disorders is further substantiated by studies revealing a decrease in amygdala reactivity towards threat-inducing experimental paradigms following successful pharmacological and non-pharmacological treatment [54], [55]. Furthermore, it has been shown in specific phobia patients that exposure therapy can reduce amygdalar hyper activation [56].

In this study, we addressed the question of neural reactivity and potential habituation in the emotion processing circuitry of SAD patients and healthy controls by employing a facial emotion discrimination paradigm that is known to stimulate activation within the amygdala and orbitofrontal regions [57]. We used this well-established stimulation protocol in lieu of subjecting SAD patients to an unbearable social threat situation. Our main goal was to examine differences in the BOLD response and its temporal dynamics within the emotion processing circuitry in SAD patients compared to healthy controls, which could explain their improper anxiety management under social stress.

## Methods

### Participants

19 social anxiety disorder patients (1 exclusion due to positive drug screening, 1 exclusion due to unacceptable image distortions within the orbitofrontal cortex caused by a dental implant, 1 exclusion due to hardware error, and 1 exclusion due to non-compliance with our study protocol) and 17 healthy subjects (2 exclusions due to non-compliance with our study protocol) were recruited from the local community via billboard announcements. Thus, 15 patients (7 male/8 female, mean age (SD): 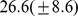 years) and 15 healthy subjects (8 male/7 female, mean age (SD): 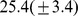 years) were included in the final data analysis.

Before inclusion, all volunteers were clinically assessed by a trained psychiatrist at the Department of Psychiatry of the General Hospital in Vienna. This examination included a general physical and neurological screening and medical history assessment. Psychological status was evaluated using the German version of the Structured Clinical Interview for DSM-IV Diagnosis (SCID), Hamilton Anxiety Rating Scale (HAM-A), Spielberger State-Trait Anxiety Inventory for Adults (STAI), and Liebowitz Social Anxiety Scale (LSAS). A summary of the psychometric scores is provided in Table 1.

**Table 1:** Psychometric assessment of participants.

	Age	HAM-A	STAI (state)	STAI (trait)	LSAS
SAD patients	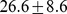	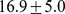	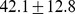	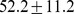	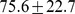
Healthy volunteers	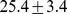	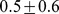	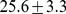	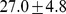	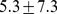
p (two-tailed)	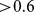	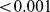	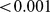	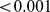	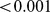

Hamilton Anxiety Rating Scale (HAM-A), Spielberger State-Trait Anxiety Inventory for Adults (STAI), and Liebowitz Social Anxiety Scale (LSAS) were used to evaluate psychiatric status and quantify severity of anxiety symptoms. Table shows mean scores 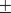 standard deviation of SAD patient and healthy control group.

Inclusion criteria for all subjects were physical health, signed written informed consent and age of 18 to 50 years. In addition, patients had to fulfill criteria for social anxiety disorder according to DSM-IV criteria assessed by the SCID. Exclusion criteria were any peculiarities in the physical and neurological assessment, pregnancy and any former or current psychiatric DSM-IV diagnosis, except SAD in the patient group. All subjects had to be free of any psychotropic medication within the last three months, free of current drug use, and had to be without past periods of substance abuse. Absence of current substance abuse was ensured by a compulsory drug screening at the day of the measurement using ToxiQUICK PAN-10 test panels (ACON Laboratories, San Diego, USA).

All subjects were financially reimbursed for their participation. The study protocol was approved by the institutional review board of the Medical University of Vienna.

### Emotion Discrimination and Object Discrimination Task

**Figure 1:**
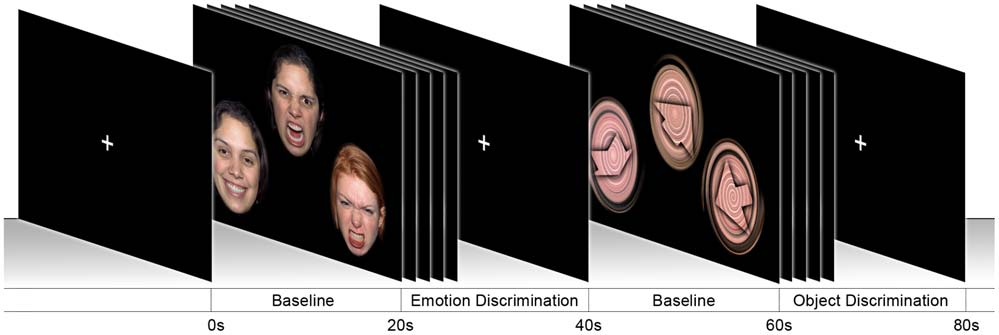
Experimental paradigm. Facial emotion and object discrimination tasks were presented in alternating individual blocks for 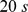. Between task conditions, a white fixation cross was presented for 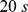 to serve as a baseline condition. Each task block was repeated five times, yielding a total paradigm length of 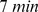. The faces, displayed in Figure 1, were obtained from the NimStim facial stimulus set [111]. We informed Nim Tottenham about our submission and obtained written consent to use it under the license model of PLoS ONE.

Subjects performed a facial emotion discrimination task (EDT) including a control condition using object discrimination (ODT) introduced by [57] and described in detail by our group in [58]. The stimulus material of the object discrimination task was slightly modified to reduce activation differences in primary visual areas between conditions (Figure 1).

The two discrimination tasks were presented alternatively to our subjects in a blocked design, with five 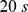 blocks of each task condition and 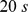 fixation cross baseline condition in-between, at the beginning and at the end of the run (resulting total length: 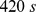). In the EDT condition participants were presented with a triplet of faces expressing one of seven emotions (anger, disgust, fear, happiness, sadness, surprise, or calmness) [59]. They were instructed to select which of the two emotional faces presented left and right at the bottom of the screen matches the target face at the top, by pressing either the left or the right button of their MR-compatible response pad. The ODT was designed likewise, but the faces were replaced by contours of geometrical shapes superimposed on a skin-colored background. Subjects were told to focus on the task and react as quickly as possible, but no explicit time limit was given for their decisions.

Individual stimuli were taken from a set of 100 different EDT and 50 ODT combinations and presented in true randomized order using Presentation (Neurobehavioral Systems Inc., San Francisco, CA, USA) projected to a semi-transparent screen located at the back end of the scanner bore. EDT stimuli were designed using the NimStim Set of Facial Expressions (MacArthur Foundation Research Network on Early Experience and Brain Development; http://www.macbrain.org/resources.htm). Participants were verbally instructed before the scan to fully understand the paradigm, without exposure to the stimulus material. Two-sample t-tests were used to compare task accuracy and response time between groups.

### Data Acquisition

MRI measurements were performed on a 3 Tesla Tim Trio MR scanner (Siemens Medical, Erlangen, Germany). Subjects were scanned using the manufacturer's 32-channel head coil. 225 whole-brain volumes (matrix size: 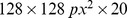 slices) were obtained at a repetition time of 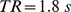 employing a single-shot echo planar imaging (EPI) sequence (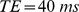, 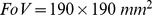, 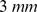 slice thickness, 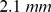 inter-slice gap, and 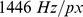 bandwidth). Note that the resulting voxel size was below 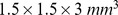 in order to reduce MRI signal losses in ventral brain regions caused by intra-voxel dephasing effects due to local magnetic field inhomogeneities [60].

A series of 5 repetitive MR excitations (dummy scans) were used before the actual data acquisition to ensure steady-state data. To get adapted to the MR environment, all participants performed a simple sensory-motor task (cued bimanual finger tapping) before the actual fMRI task.

### Pre-processing and Data Analysis

Acquired fMRI data were pre-processed and analyzed in SPM8 (FIL Methods Group, Wellcome Trust Centre for Neuroimaging, University College London; http://www.fil.ion.ucl.ac.uk). Preprocessing included correction for slice-timing differences [61], realignment to compensate for bulk head motion, segmentation [62], normalization to standard MNI space (at 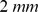 isotropic voxel size), and spatial smoothing with an isotropic Gaussian kernel of 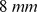 FWHM.

First-level single-subject analysis was conducted using the general linear model (GLM) framework provided by SPM8. Stick functions of the onsets of the two task conditions were convolved with SPM8's canonical hemodynamic response function. These two separate regressors were designed to model changes in hemodynamic responses during emotional face discrimination and object discrimination, respectively. Further, all six realignment parameters obtained from preprocessing were included in the design matrix to reduce residual motion effects.

Second-level group analysis was performed by conducting a whole-brain ANOVA using the individual contrast for activation differences between emotion and object discrimination (i.e. 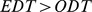) and including age, gender, and LSAS score as covariates (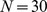). This design was used to identify task-related changes in brain activation consistent at inter-subject level. Significance threshold was set to 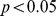 (FWE whole-brain corrected for multiple comparisons) and a minimum cluster size of 75 voxels (equates to 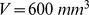) was chosen.

Regions that showed task-related activations were further analyzed for time-dependent changes. We performed GLM analyses for each subject by modeling each task block individually. As in the first-level analysis, realignment parameters were also included as nuisance regressors. This analysis yielded 10 individual parameter estimates of interest for each subject (5 blocks EDT, 5 blocks ODT) to allow for assessing habituation effects during the experiment. Spherical ROIs (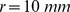) were defined around the peak voxels revealed in the second-level group analysis (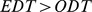). Percent signal change was calculated based on the individual beta values extracted from the single-subject analysis for each ROI and task block. Linear regressions, as implemented in MATLAB, on the group averages of the task-related signal changes were used to model temporal habituation effects with a threshold of 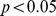. To verify the observed habituation effects, we also conducted a repeated-measures ANOVA on the single-subject parameter estimates (subject by task block) for all task-active ROIs.

## Results

### Behavioral Data

No statistically significant group differences were found regarding task accuracy (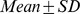; EDT: 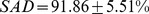, 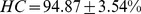; ODT: 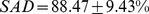, 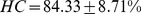) and response time (EDT: 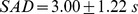, 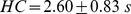; ODT: 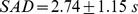, 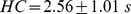).

### fMRI Data

#### Task-related Effects

**Figure 2:**
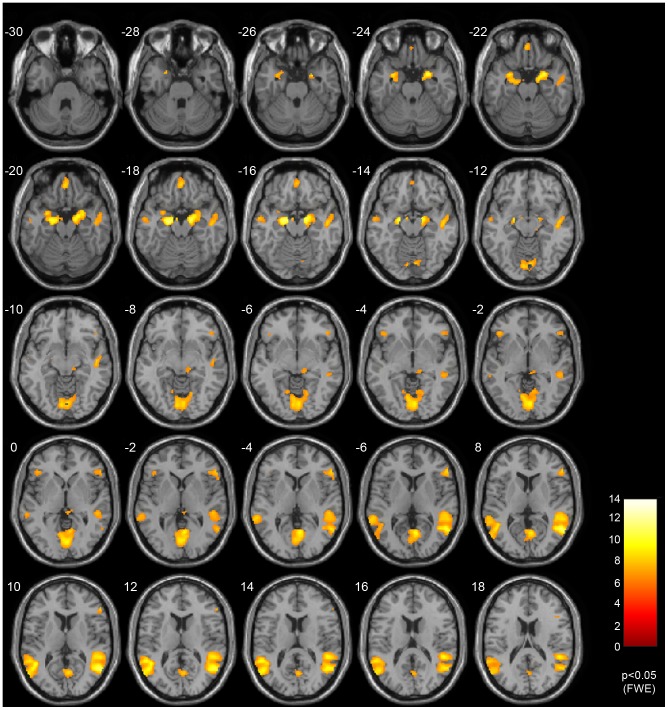
SPMs of task-related effects. Second-level ANOVA (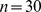 subjects, 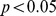 FWE corrected, 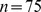 voxels minimum cluster size) was employed to identify task-related networks. Contrast of interest was emotional face discrimination vs. object discrimination. Statistics and coordinates of significant clusters are shown in table 2.

The main result of the second-level ANOVA is displayed in Figure 2 (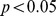 FWE corrected, minimum cluster size 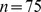). Our contrast of interest was the difference between facial emotion processing and object discrimination tasks. The following clusters were identified: left and right temporal gyrus, posterior cingulate cortex, left and right amygdala, orbitofrontal cortex, left and right dorsolateral prefrontal cortex, right middle frontal cortex, and the pulvinar part of thalamus (please refer to table 2 for details). None of the covariates age, gender, and LSAS score revealed any significant effects in our whole-brain analysis when properly correcting for multiple univariate test. For this dataset, we previously reported hyper activation of the OFC in SAD patients when compared with healthy controls [63]. This difference, however, was only marginal as it did not survive FWE correction and can be explained within the context of the observed habituation effects.

**Table 2:** Task-related effects.

Region		Coord. [mm]			Peak Level	
Anatomy	BA	x	y	z	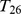	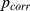
right temporal gyrus	20	54	−58	8	11.08	0.000
left temporal gyrus	20	−46	−54	10	9.79	0.000
right amygdala	34	16	−8	−16	10.87	0.000
left amygdala	34	−18	−12	−18	11.52	0.000
orbitofrontal cortex	11	2	48	−18	8.42	0.000
right inf. and dorsolat. PFC	45–47	56	34	8	8.26	0.000
left inf. and dorsolat. PFC	45–47	−42	30	2	7.42	0.003
right middle frontal gyrus	6	46	2	58	7.85	0.000
PCC, Cuneus	31	6	−64	4	9.45	0.000
pulvinar thalamus		6	−32	0	6.86	0.009

Second-level ANOVA revealed eight regions significantly more active (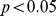 FWE corrected, 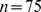 voxel minimum cluster size) in the emotional face discrimination task compared to the object discrimination task. These task-relevant areas were used as regions of interest for the subsequent assessment of group-related effects. Atlas information and corresponding Brodmann areas (BA) taken from Talairach-Tournoux Atlas. *T*-values for 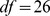 degrees of freedom.

#### Social anxiety disorder related habituation effects

**Figure 3:**
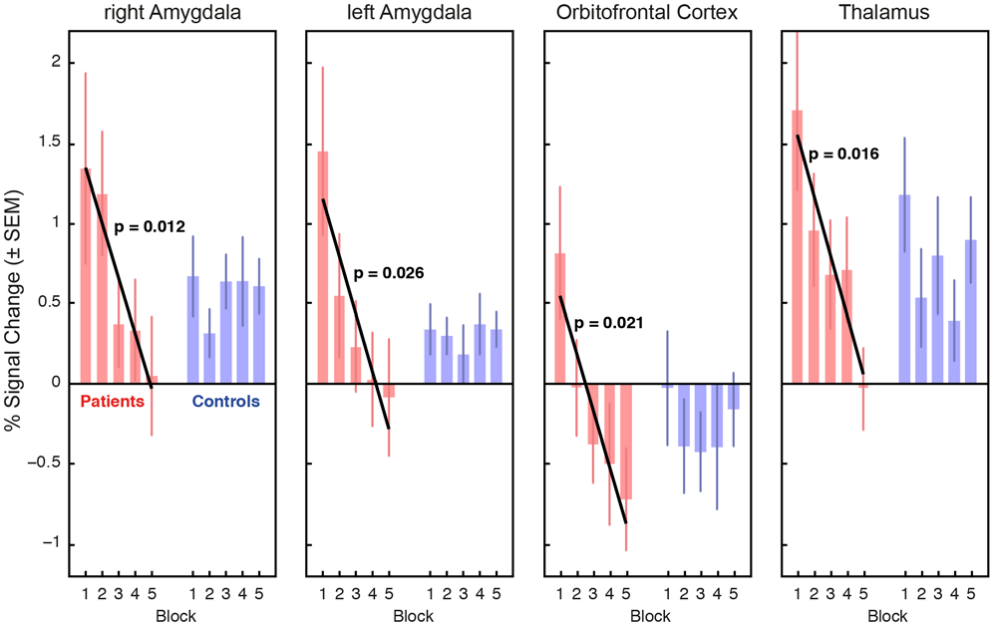
Habituation effects within amygdalae, orbitofrontal cortex and thalamus. When comparing mean BOLD responses of individual emotion discrimination task blocks, patient group (red bars) showed significant adaptations towards emotional stimuli, which are not observed in the control group (blue bars).

Subsequently, we performed linear regression analysis within our task-related regions to test for time-dependent adaptations. In SAD patients we observed a significant linear decrease in bilateral amygdalae, orbitofrontal cortex, and pulvinar activation (all 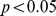) over the five emotion discrimination task blocks (Figure 3). No such effects were found for healthy controls. For both groups control conditions (ODT) did not show significant habituation effects.

In full agreement with these findings, our repeated-measures ANOVA of the same data revealed significant time effects exclusively for the SAD group in the regions highlighted before: rAmy (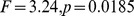), lAmy (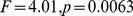), pulvinar (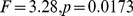), OFC (F = 5.93, p = 0.0005).

**Figure 4:**
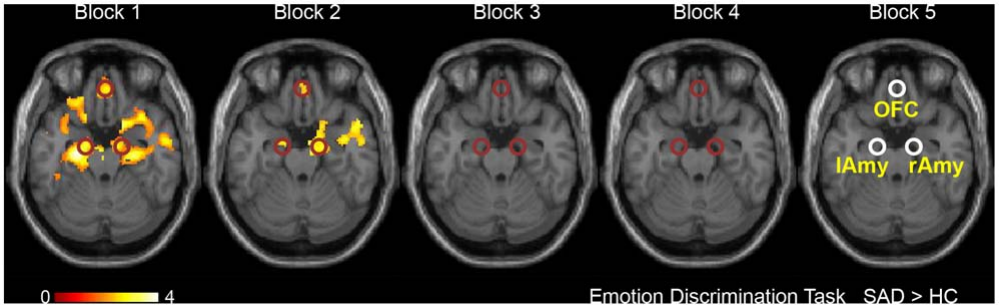
SPMs of habituation effects within the limbic system and orbitofrontal cortex. Peak voxels of clusters showing task-related differences (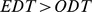, 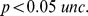 for illustration purpose, cf. Figure 2) were used as center voxels for ROIs (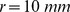), which are shown in the map of block 5. The presented slice corresponds to slice 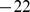 in Figure 2.

For illustrative purposes we also performed two-sample t-tests between SAD patients and controls for each of the five EDT blocks. Figure 4 shows the corresponding statistical maps. It can be seen that statistically significant activation differences are found only in the first and second blocks. Together with figure 3 this clearly indicates habituation in SAD patients.

## Discussion

In this study, task-related (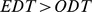) activations were observed not only in both right and left amygdalae, but also in left and right dorsolateral prefrontal cortex, which have been linked to voluntary cognitive control and performance monitoring [64]. In addition, activation was found within the medial orbitofrontal cortex, which is a critical area for modulating fear [65], [66]. All these areas are part of the cortico-limbic anatomical and functional network described in detail by [30].

When comparing time-dependency of the neural response between SAD patients and healthy volunteers, habituation was observed exclusively in patients and only for the emotion discrimination task. More specifically, the amygdalae, OFC and thalamus showed a significant linear decrease in activation. No corresponding habituation effects were found in healthy controls. Further analysis revealed that significantly higher activation in SAD patients compared to controls was present only during the first blocks of the experiment, when task and nature of the presented stimulus material were truly novel to the volunteers (see Figure 3). Note that participants were nave to the stimulus material. As experience with the emotion discrimination task increased, this hyperactivation decreased as an expression of gradual habituation of the implicated anxiety network. This result is compatible with an the assumption that increased effort is needed within modulatory networks in prefrontal brain areas of SAD patients to exert sufficient top-down control over hyperactivation in the amygdala when confronted with unknown and potentially threatening stimuli. This interpretation is further substantiated by the concurrent habituation in the pulvinar region of the thalamus that was found in, and only in, the SAD patient group. The pulvinar is a well-known key structure within the attention control network and crucial when coping with distractions [67], [68]. In humans, BOLD increase in the pulvinar has been observed when a subject consciously perceives a stimulus [69].

A previous behavioral study where participants were required to give several impromptu speeches compared arousal levels between high and low socially anxious subjects [70]. There it was shown that less anxious subjects exhibited significant reduction of arousal across the experiment, while no such reduction was observed in high anxious subjects. The paradigm employed in the present study, however, was designed to address the specific deficits of SAD patients not during anticipation, but in an actual performance situation that requires the proper functioning of the amygdala-prefrontal network, an assumed regulation network for affective states that is putatively disturbed in patients. As a consequence, it may be deduced that the control group in this study did not feel unusual, unpleasant or inappropriate arousal levels and thus no habituation was required. SAD patients, on the other hand, might have experienced the face matching task, the inevitable confrontation with human faces, their own (potentially negatively biased) interpretation of the perceived emotions, or a combination of all these factors intimidating and fear-inducing. This interpretation would be compatible with findings from similar experiments where habituation in the amygdala was predominantly the consequence of repeated presentation of fear and threat cues [48]–[52], which could explain why habituation of the BOLD response was only found in patients and not observed as a physiological feature in healthy controls.

Furthermore, it has been shown that diverting attention suppresses amygdala responses to human faces [71]. The congruent activation time course of the pulvinar thalamus could be associated with a form of attention shift away from the emotionally unsettling features of the presented content. Anxiety has been extensively linked to avoidance and emotional neural systems might functionally and anatomically overlap with motivational systems [72]–[78] and this form of attentional diversion or withdrawal might represent a cognitive avoidance strategy.

Our results add further evidence for a preservation of a neurobiological adaptive potential in SAD patients in situations that do not trigger full symptom provocation. Therefore, in these situations with low and medium anxiety intensity, successful top-down control mechanisms of prefrontal areas could be available even for SAD patients to maintain a balanced affective state. Thereby, a conclusive model may be nurtured where initial top-down control of prefrontal areas over amygdala hyperactivation in situations with lower and medium fear intensity achieves to maintain a balanced state. This might even lead to a gradual habituation of cortical and subcortical key structures of fear processing. However, when increasing the degree of intensity or the degree of fear-related content, subcortical activation might exceed cortical regulation capabilities, leading to symptom provocation and withdrawal of a specific situation before adaptive mechanisms might become effective.

Our data provides two important findings. First, we observed a continuous significant linear decrease in amygdala activation from the first to the last block specific to SAD patients. This buttresses results derived from a similar emotional faces processing paradigm (gender matching task as described in Stein et. al. [28]) where temporal adaptation of the BOLD signal in the amygdalae of SAD patients has been found [79]. They further hypothesized that activation or habituation differences in additional parts of the emotion regulation circuitry might be fundamental in the pathophysiology of SAD. As a second finding, our results for the first time provide evidence that the OFC indeed shows habituation effects similar to amygdala regions. It may thus be suggested that habituation is a phenomenon that not only affects the central hub of the emotion processing circuitry (i.e. the amygdalae) but also brain regions known for their modulating function, therefore favoring models including networks in the prefrontal cortex.

Past studies have provided converging evidence that the amygdala is a key structure for the evaluation and processing of emotionally relevant information in humans, as well as other mammals [80]–[83]. Notably, amygdala functions have been linked to risk avoidance and fear phenomenology [51], [84]–[86]. The amygdala is, however, only one part of the larger regulatory network connecting to regions of the prefrontal cortex [30]. In social cognition, the amygdala plays a central role in social reward anticipation and processing of ambiguity [87]. Consistent with these findings, amygdala involvement has been outlined as central in the pathophysiology of social anxiety disorders [27], [88]. A number of studies have investigated the processing of emotional faces in social anxiety disorder and identified and retrieved hyperactivity of the amygdala in response to negatively valenced (harsh or angry) and neutral in SAD patients compared to healthy volunteers [28], [89], [90]. As such, a concept of exclusively bi-unique connection between threat and amygdalar function may be too simple, as the amygdala has also been found active when processing other emotional valences and, presumably, has a broader, more universal role in mental processes [91], [92].

Multitudinous studies have outlined well-established anatomical amygdala-prefrontal networks in rodents [93], [94] and non-human primates [95]–[97] using invasive investigation methods, which have been successfully translated to humans using diffusion-tensor imaging (DTI) [98]. Functional relationship has been outlined by an increasing number of electro-physiological [65] and imaging studies [99], [100]. Noteworthy, this pathway mediates fear extinction [101], as well as perception and assessment of threat signals [102] but also comprehension of other emotional valences [103], [104].

Anatomical alterations of the amygdala-prefrontal network have been repeatedly reported in anxiety disorders [105] and SAD in particular [44]. Recently, reduction in functional connectivity between the orbitofrontal cortex and the amygdala has been observed in SAD patients [39], [41]. In stressful situations, such as public speaking [106], [107] or other anxiety provoking circumstances [108], (social) anxiety disorder patients present with reduced activation of the OFC, suggesting failed fear suppression. This study used emotional faces to provoke activations within the emotion processing network. While sensitivity towards emotional faces has been reported before [27], this paradigm was not designed to put the patients in an actual stress situations where they would fully experience symptoms of their anxiety disorder. While not in a social threat situation, SAD patients showed concurrent habituation of the medial orbitofrontal cortex and bilateral amygdalae. We interpret this as an initially increased effort in down-regulating amygdalar activation by the orbitofrontal cortex. In more stressful situations, SAD patients could fail to successfully recruit these essential regulatory areas.

We are well aware that the proposed model might be too simplistic as it fails to include a multitude of regulatory influences both on a molecular and on a network level. For example, besides cortical top-down control of subcortical regions, bottom-up influences of the amygdala on prefrontal regions should also be considered. This has been shown in an animal model of conditioned fear, providing evidence for a significant influence of the basolateral amygdala on neural activation in the medial prefrontal cortex [109].

Here we emphasized the importance of the amygdala regulation circuitry in the pathophysiology of SAD. While, undoubtedly, the amygdala plays a crucial role in anxiety disorders and emotion processing, our findings suggest involvement of the OFC in the neuro pathophysiology of SAD. Interestingly, only few studies report OFC activation or differences in SAD patients. It might be suggested that this could be caused by the strong susceptibility artifacts in ventral brain areas at high magnetic fields, which make detection of neural activation in these regions particularly challenging. Here we used well-established optimized MRI sequences to robustly acquire data from these areas, as well.

Our study suggests dysfunctions within the emotion processing and regulation network in SAD, in particular with respect to regulation latencies. Therefore, we stress the importance of further studies with advanced analysis methods, such as dynamic causal modeling [110], that enable description of the temporal and causal relationships within the highlighted network. For clinical applications, basic research on this network could provide validation for neurobiological models, inspiration for new therapeutic methods, and, assuming further methodological advancements, support in diagnosis, prognosis and treatment progress.
